# MUC5B regulates goblet cell differentiation and reduces inflammation in a murine COPD model

**DOI:** 10.1186/s12931-021-01920-8

**Published:** 2022-01-18

**Authors:** Xuan Huang, Weijie Guan, Bin Xiang, Wei Wang, Yanqing Xie, Jinping Zheng

**Affiliations:** State Key Laboratory of Respiratory Disease, National Clinical Research Center for Respiratory Disease, Guangzhou Institute of Respiratory Health, First Affiliated Hospital of Guangzhou Medical University, No 151 Yanjiang Road, Guangzhou, 510120 People’s Republic of China

**Keywords:** *MUC5B*, COPD, SPDEF, Goblet cell differentiation, Inflammation

## Abstract

**Background:**

Airway mucus hypersecretion is one of the important pathological features of chronic obstructive pulmonary disease (COPD). MUC5B is the main mucin expressed in the airways of COPD patients and has been indicated to play an important role in airway defense. However, the specific biological function of MUC5B in COPD and the possible mechanism are not clear.

**Methods:**

We established a COPD model with 24-week-old MUC5B^−/−^ mice exposed to cigarette smoke and tested our hypothesis through lung function tests, HE and PAS staining, immunohistochemistry (IHC), western blot, q-PCR and ELISA.

**Results:**

Compared with MUC5B^+/+^ mice, MUC5B^−/−^ mice had worse general condition and lung function, increased inflammatory infiltration, reduced goblet cell differentiation as indicated by decreased PAS staining (PAS grade: 1.8 ± 0.24 vs. 0.6 ± 0.16), reduced MUC5AC expression (ELISA: 0.30 ± 0.01 vs. 0.17 ± 0.01 mg/ml, q-PCR: 9.4 ± 1.7 vs. 4.1 ± 0.1 fold, IHC score: 3.1 ± 0.9 vs. 1.6 ± 0.7), increased macrophage secretion of inflammatory factors (TNF-α and IL-6) and expression of downstream pathway factors (ERK1/2 and NF-κB), decreased expression of SPDEF and STAT6, and increased expression of FOXA2.

**Conclusion:**

The protective effect of MUC5B in the development of COPD was mediated by the promotion of goblet cell differentiation and the inhibition of inflammation. The role of MUC5B in regulating inflammation was related to macrophage function, and goblet cell differentiation was promoted by the induced expression of STAT6 and SPDEF. This study describes a mechanism of mucus hypersecretion and identifies MUC5B as a new target for the treatment of mucus hypersecretion.

**Supplementary Information:**

The online version contains supplementary material available at 10.1186/s12931-021-01920-8.

## Background

Chronic obstructive pulmonary disease (COPD) is a disease characterized by persistent respiratory symptoms and airflow limitations. The main characteristics are dyspnea, chronic cough, expectoration, wheezing and other symptoms. The latest epidemiological data show that the total prevalence of COPD in Chinese adults is approximately 100 million [1]. The direct medical cost of COPD is as high as 89 billion yuan, ranking first in China’s disease burden, and COPD is becoming a serious public health problem [2].

Mucus hypersecretion is one of the important pathophysiological features of COPD. In COPD patients, long-term stimulation, such as smoking or biofuel exposure, induces pathological changes, such as goblet cell differentiation and mucous gland hypertrophy. Mucus obstruction caused by mucus hypersecretion is common in COPD patients and is associated with decreased lung function and acute exacerbation [3, 4]. However, the mechanism of mucus hypersecretion in COPD is not clear, and treatment has not received attention.

Airway mucin is mainly composed of the gel-forming mucins MUC5AC and MUC5B. MUC5AC is mainly expressed in asthma patients [5–7], whereas MUC5B is mainly expressed in COPD patients [7–10]. Studies by Roy MG et al. showed that MUC5B plays an important role in airway defense [11]: in MUC5B^−/−^ mice, mucosal ciliary clearance function was decreased, apoptotic macrophages were increased, and IL-23 level was significantly decreased. Recent studies indicate that MUC5B may be related to the secretion of airway mucus; MUC5B^−/−^ mice produce almost no mucus, and Muc5b contributes extensively to mucus obstruction [12, 13], which seems to contradict the protective effect of MUC5B in the airway. Goblet cells are the main source of airway mucus, and SAM-pointed domain-containing Ets-like factor (SPDEF) plays an important role in the differentiation of airway goblet cells [14]. Studies have shown that SPDEF can regulate the expression of MUC5B in the airway, and SPDEF^**−/−**^ mice have phenotypes similar to those of MUC5B^**−/−**^ mice [15]. Therefore, we speculate that MUC5B can promote the expression of SPDEF and thereby promote the differentiation of goblet cells, leading to mucus hypersecretion. This study was designed to identify the role of MUC5B in the development of COPD and the mechanism by establishing a COPD model in MUC5B^−/−^ mice.

## Methods

### Establishment and identification of MUC5B^−/−^ mice

MUC5B knockout mice were constructed by embryonic stem (ES) cell targeting and confirmed by PCR. Details are provided in Additional file 1.

### Animal groups and COPD modeling

Cigarette smoke exposure can mimic the occurrence and development of COPD [16]. Male C57B6J and MUC5B^−/−^ mice aged six to eight weeks were randomly divided into 4 groups (n = 15 in each group of MUC5B^+/+^ mice and n = 10 in each group of MUC5B^−/−^ mice): a MUC5B^+/+^ control group, a MUC5B^−/−^ control group, a MUC5B^+/+^ smoke-exposed group and a MUC5B^−/−^ smoke-exposed group. A custom-made smoking box was used for cigarette smoke exposure. Mice in the smoke-exposed group were placed into the smoking box, which was 60 × 57 × 100 cm. (The oxygen concentration in the box was 18–20% during smoke exposure.) Two vents with a diameter of 1 cm were located on the top of the box. Initially, the total particulate matter concentration in the box was 741.4 mg/m^3^, the respirable gas particle concentration was 34.6 mg/m^3^, the oxygen content was greater than 20%, the CO_2_ concentration was 4000 ~ 5000 ppm, and the CO concentration was 500 ~ 800 ppm. A cigarette was connected to a cigarette holder, and smoke from the cigarette was continuously aspirated with a 60 ml syringe and injected into the box through an infusion tube connected to a three-way tube (Hongmei Brand, Guangdong Cigarette Factory). Each cigarette contained 11 mg tar and 13 mg smoke carbon monoxide. Smoke injection was conducted 2 times each day for 2 h each time, with 9 cigarettes used each hour, for 6 days each week. The entire model establishment process spanned 24 weeks. The animals were free to eat and drink in the smoke box during smoke exposure. A control group was exposed to normal air under the same conditions described above. During the experiment, the general condition of the mice was observed, and the body weights were measured every 2 weeks. Relevant testing was carried out at the end of the experiment.

### Lung function

Mouse lung function was analyzed using an invasive mouse pulmonary function system as described by Vanoirbeek JA et al. [17]: after system calibration, all mice were anesthetized, tracheostomized, and placed in a forced pulmonary maneuver system (Buxco Research Systems, USA). Functional residual capacity (FRC), airway resistance (RI), forced expiratory volume in 100 ms (FEV100) and dynamic compliance (Cdyn) were measured.

### Bronchoalveolar lavage (BAL) fluid extraction and cell counting

Extraction of alveolar lavage fluid was performed as described in Livraghi A et al. [18]. After lung function measurement, sterile PBS was instilled into the right lung while the left main stem bronchus was ligated. The volume of PBS was determined by the formula: (mouse weight (g) × 0.0175 ml = ml PBS instilled). BAL was performed by gently injecting and retrieving the instilled PBS three times. This procedure was repeated with an equal volume of PBS, and the fractions were pooled. The return volume was consistently > 80% of the instillation volume. BAL cells were pelleted by centrifugation at 1000×*g* for 5 min at 4 °C, and the cell-free supernatant was collected and stored at − 80 °C for further analysis. BAL cells were resuspended in 100 μl of PBS, and the total number of cells was counted with a hemocytometer (thermo Countess 3 Countess III). Cytospin slides of 30,000–60,000 cells/slide were obtained, air dried, and stained with modified Giemsa stain for differential cell counts of at least 200 cells per slide.

### Lung tissue pathology

After the extraction of BAL fluid, the right main bronchus was ligated, and the left lung was perfused with a custom-made perfusion device using 4% paraformaldehyde to inflate the lung [19]. The left main bronchus was ligated, and the left lung was removed and placed in 4% paraformaldehyde. After 24 h, the lung was removed, and the residual paraformaldehyde was rinsed carefully with PBS. The specimen was subjected to gradient dehydration and embedded in paraffin. Wax block embedding and subsequent hematoxylin–eosin (HE) staining and periodic acid-Schiff (PAS) staining were performed by the Department of Pathology of the Guangzhou Institute of Respiratory Health. The stained tissues were evaluated under a digital pathology scanner (PRECICE 500B, Unic-tech). Quantitative analysis was performed as described previously [20, 21]. The severity of peribronchial inflammation was quantified from 0 to 5 in the lung images stained by HE [21], where 0 = no cells, 1 = a few cells, 2 = a ring of cells one layer deep, 3 = a ring of cells two layers deep, 4 = a ring of cells three to four layers deep, and 5 = a ring > 4 cell layers deep. Goblet cell differentiation in the central and peripheral airways was examined on AB-PAS-stained sections. The degree of AB-PAS positive staining in each airway was semiquantitatively determined using the following five-stage grading system [20]: grade 0, no AB-PAS staining; grade 1, 25% or less of airway epithelium with AB-PAS staining; grade 2, 26–50% of airway epithelium with AB-PAS staining; grade 3, 51–75% of airway epithelium stained with AB-PAS; and grade 4, > 75% of airway epithelium with AB-PAS staining. Each slide was examined by a pathologist who was blinded to the treatment. Images of stained cells were evaluated at 200 × magnification. At least six bronchioles selected at random were evaluated in each slide, and the average score was calculated.

### Immunohistochemistry (IHC)

Lung sections were probed with primary antibodies (against SPDEF (ab53881, Abcam), STAT6 (ab44718, Abcam), FOXA2 (ab108422, Abcam), MUC5AC (ab24071, Abcam), and MUC5B (ab77995, Abcam)) and detected using an SABC kit (SA1020, Boster) according to the manufacturers’ protocols. The stained tissues were evaluated under a digital pathology scanner (PRECICE 500B, Unic-tech). ImageJ and IHC Profiler were used for quantitative analysis. IHC score was calculated as follows: (Number of pixels in zone) × (Score of the zone)/Total number of pixels in the image. The proportion of nuclear-positive staining cells was scored for analysis as follows: high proportion = 4, moderate proportion = 3, low proportion = 2, and negative staining = 1.

### ELISA

The following mouse BALF ELISA kits were used: IL-6 (DY406-05, R&D), KC (DY453-05, R&D), TNF-α (DY410-05, R&D), MUC5AC (E-EL-M0799c, Elabscience), and MUC5B (E-EL-M0800c, Elabscience). Analyses were performed according to the kit instructions.

### Lung tissue protein extraction and western blotting

Lung protein extraction was performed with tissue lysis buffer (RIPA buffer, P0013B, Beyotime; phosphatase inhibitor cocktail 2, P5726, phosphatase inhibitor cocktail 3, P0044, Sigma). Then, the protein concentration was determined using a BCA kit (23227, Thermo), and primary antibody solutions diluted with 2.5% BSA (Amresco) (ERK1/2: 1:2,000, CST, 9102S; p-ERK1/2: 1:2,000, CST, 9102S; p-NF-κB: 1:2,000, Abcam, ab28856; GAPDH: 1:8,000, Abcam, ab181602; SPDEF: ab53881, Abcam; STAT6: ab44718, Abcam; and FOXA2: ab108422) and secondary antibodies (goat anti-rabbit, 1:5000, Proteintech) were used. ImageJ software was used for grayscale analysis.

### Lung tissue RNA extraction and qPCR

Lung tissue RNA was extracted using TRIzol reagent (Invitrogen, 15596018). Reverse transcription was carried out using a reverse transcription kit (Takara, RR047A). The primer sequences of the genes of interest were designed using primer design software, and primer specificity was determined based on the BLAST alignment in NCBI. The primers were synthesized by Shanghai Shenggong Bioengineering Co., Ltd., and the sequences were as follows: Muc5ac: Forward: 5′-ATGGGCTGTGTTCCTGTGTC-3′ and Reverse: 5'-CAGAACATGTGTTGGTGCAGTC-3'; Muc5b Forward: 5′-GTGAGGAGGACTCCTGTCAAGT-3′ and Reverse: 5′-CCTCGCAGAAGGTGATGTTG-3′; and 18S: Forward: 5′-GCAATTATTCCCCATGAACG-3′ and Reverse: 5′-GGCCTCACTAAACCATCCAA-3′. qPCR was performed using a fluorescent quantitative PCR kit (Bio-Rad, 172-5201AP). After the reaction, the CT value of each sample was determined. 18S was used as an internal reference, and the Muc5ac and Muc5B mRNA levels were calculated by the 2 − ΔΔCT method.

### Statistical analysis

The data are expressed as the mean ± SD or median (interquartile range). After the data were tested for normality, comparisons among groups were performed using one-way ANOVA or nonparametric tests as appropriate. A value of P < 0.05 was considered statistically significant. All analyses were performed using SPSS version 22.0 (SPSS Inc., USA).

## Results

### Characteristics of COPD in a smoke-exposed mouse model: inflammatory infiltration, impaired lung function and emphysema

Compared with the lungs of mice in the control group, those of mice in the smoke-exposed group exhibited severe emphysema and neutrophil and macrophage infiltration after cigarette smoke exposure (Fig. 2), and the expression of inflammatory factors in BAL fluid was increased in the smoke-exposed group (Fig. 2). In addition, lung function was worse in the smoke-exposed group than in the control group (as evidenced by increased RI and FRC and decreased Cdyn and FEV100 in the former) (Fig. 5). These results show that the model we established conformed to the pathological characteristics of COPD and could be used in COPD-related research.

### Goblet cell differentiation is decreased in MUC5B^−/−^ mice

**Fig. 1:**
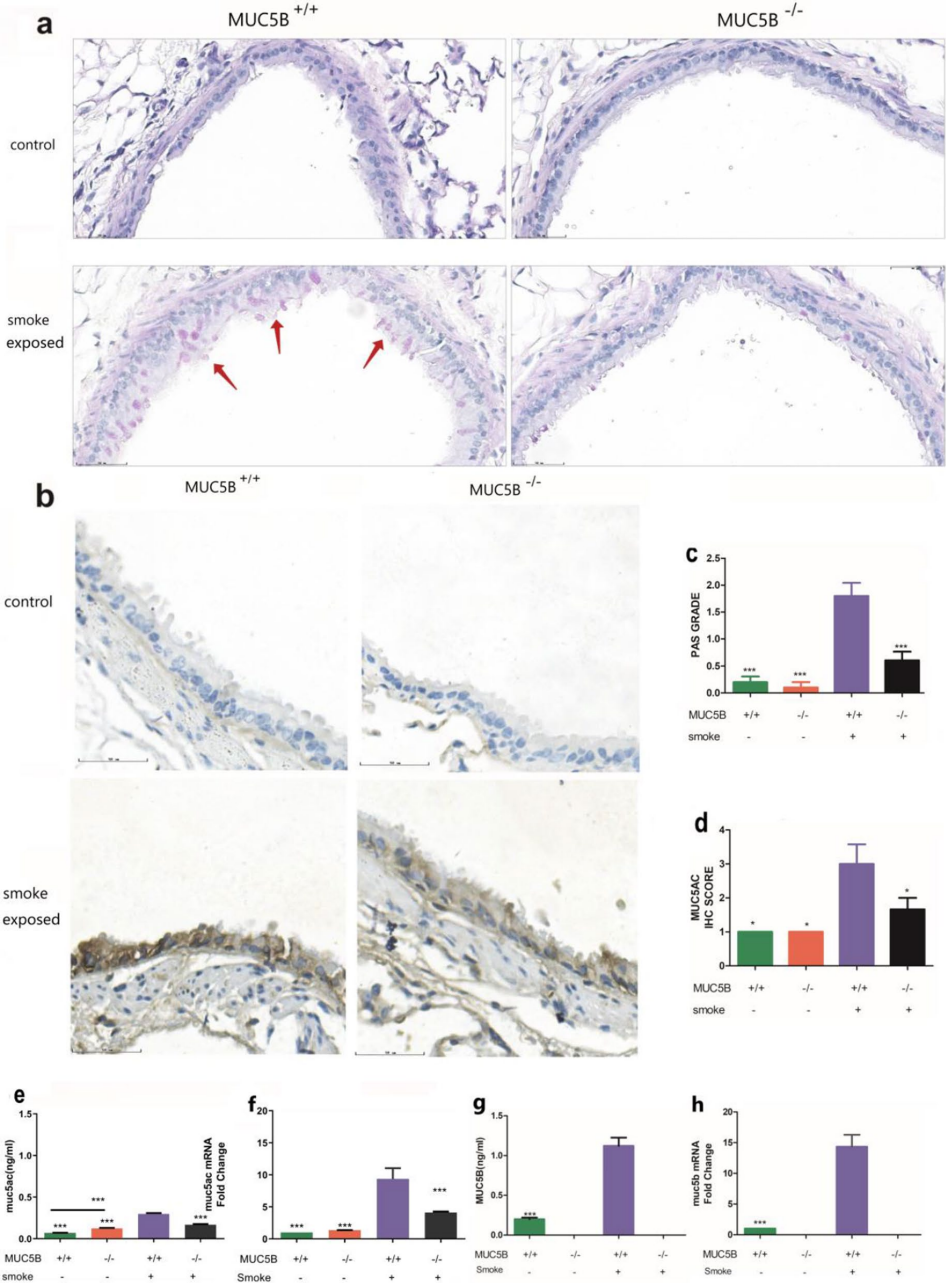
MUC5B^−/−^ mice have decreased goblet cell differentiation. MUC5B^+/+^ mice (n = 15 per group) and MUC5B^−/−^ mice (n = 10 per group). **a** PAS staining. **b** MUC5AC IHC. **c** PAS grade. **d** MUC5AC IHC score. **e** ELISA of MUC5AC in BAL fluid. **f** RT-qPCR of MUC5AC in lung tissue. **g** ELISA of MUC5B in BAL fluid. **h** RT-qPCR of MUC5B in lung tissue. “*” indicates a significant difference; ***P < 0.001, **P < 0.01 compared with smoke-exposed MUC5B^+/+^ mice

We evaluated the differentiation of airway goblet cells in terms of pathology and the goblet cell marker MUC5AC. Specific staining for goblet cell differentiation—AB-PAS staining—showed a large amount of positive staining in the airway of MUC5B^+/+^ mice and a significantly lower amount in MUC5B^−/−^ mice (P < 0.001) (Fig. 1a, c). The expression of MUC5AC protein and its RNA was higher in the smoke-exposed group than in the control group. Among mice in the control group, MUC5AC expression was higher in MUC5B^−/−^ mice than in MUC5B^+/+^ mice (P < 0.001). In the smoke-exposed group, MUC5B^−/−^ mice expressed less MUC5AC protein and mRNA than MUC5B^+/+^ mice (P < 0.001) (Fig. 1b, d, e, f). No MUC5B protein or gene expression was observed in the lungs of MUC5B^−/−^ mice (Fig. 1g, h).

### Infiltration of inflammatory cells is greater in MUC5B^−/−^ mice

**Fig. 2:**
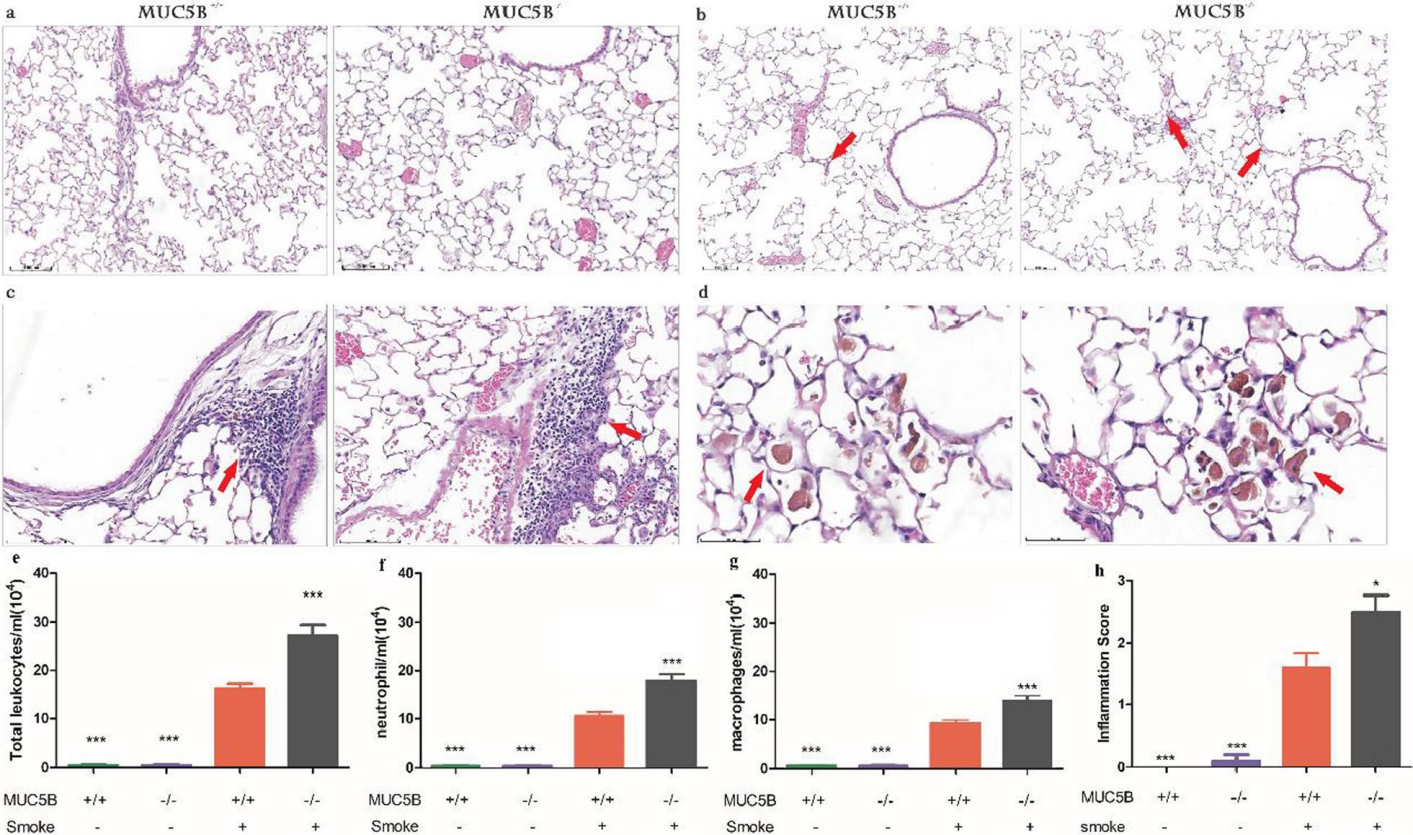
MUC5B^−/−^ mice had more severe pulmonary inflammation. **a** HE staining in the control group. **b**–**d** HE staining in the smoke-exposed group: **b** emphysema; **c** neutrophil infiltration; **d** macrophage infiltration; **e** total leukocytes, **f** neutrophils and **g** macrophages in bronchoalveolar lavage fluid (BALF). **h** inflammation score. “*” indicates a significant difference; ***P < 0.001 compared with smoke-exposed MUC5B^+/+^ mice

The results showed that the numbers of total leukocytes (Fig. 2e), neutrophils (Fig. 2c, f) and macrophages (Fig. 2d, g) in the smoke-exposed group were significantly higher than those in the control group (P < 0.001) and that in the smoke-exposed group, MUC5B^−/−^ mice had more severe inflammation than MUC5B^+/+^ mice (P < 0.001) (Fig. 2c–h).

### Decreased SPDEF expression in MUC5B^−/−^ mice

**Fig. 3:**
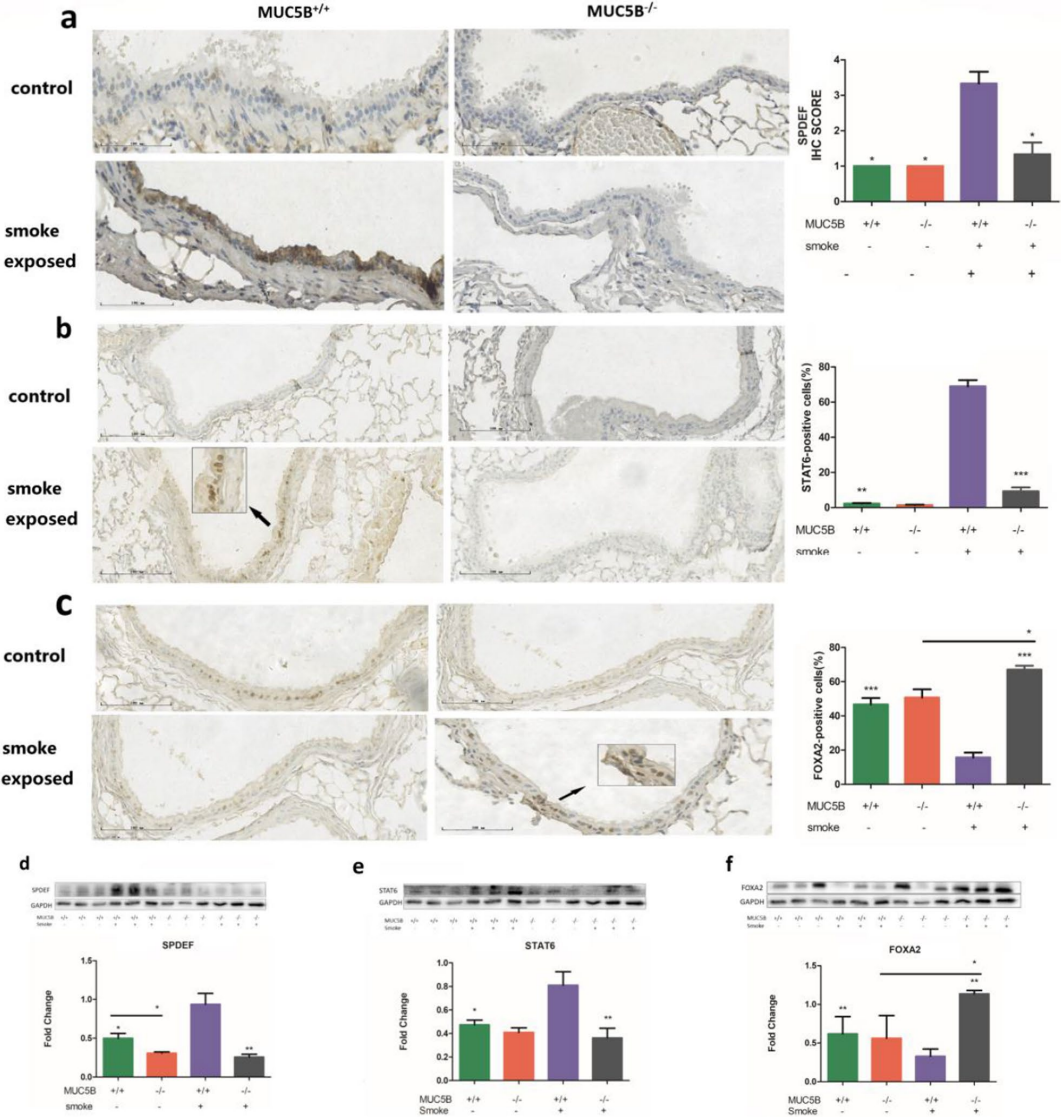
MUC5B^−/−^ mice have decreased SPDEF expression. MUC5B^+/+^ mice (n = 15 per group) and MUC5B^−/−^ mice (n = 10 per group). **a** SPDEF immunohistochemical staining and IHC score. **b** STAT6 immunohistochemical staining and positive cell percentage. **c** FOXA2 immunohistochemical staining and positive cell percentage. **d** SPDEF western blot and grayscale analysis. **e** STAT6 western blot and grayscale analysis. **f** FOXA2 western blot and grayscale analysis. “-” indicates a significant difference between two groups. “*” indicates a significant difference; ***P < 0.001, **P < 0.01, *P < 0.05 compared to smoke-exposed MUC5B^+/+^ mice

To clarify the mechanism by which MUC5B regulates goblet cell differentiation, we measured changes in SPDEF and its upstream regulatory factors, which are the core factors that regulate airway goblet cell differentiation. The control group showed low expression of SPDEF (Fig. 3a, d) and STAT6 (Fig. 3b, e) but high expression of FOXA2 (Fig. 3c, f); relatively higher SPDEF and STAT6 expression and lower expression of FOXA2 was observed in the smoke-exposed group. The expression of SPDEF and STAT6 in MUC5B^**−/−**^ mice was lower than that in MUC5B^+/+^ mice, and the expression of FOXA2 in MUC5B^**−/−**^ mice was higher than that in MUC5B^+/+^ mice.

### Increased macrophage-related inflammatory factors in MUC5B^−/−^ mice

**Fig. 4:**
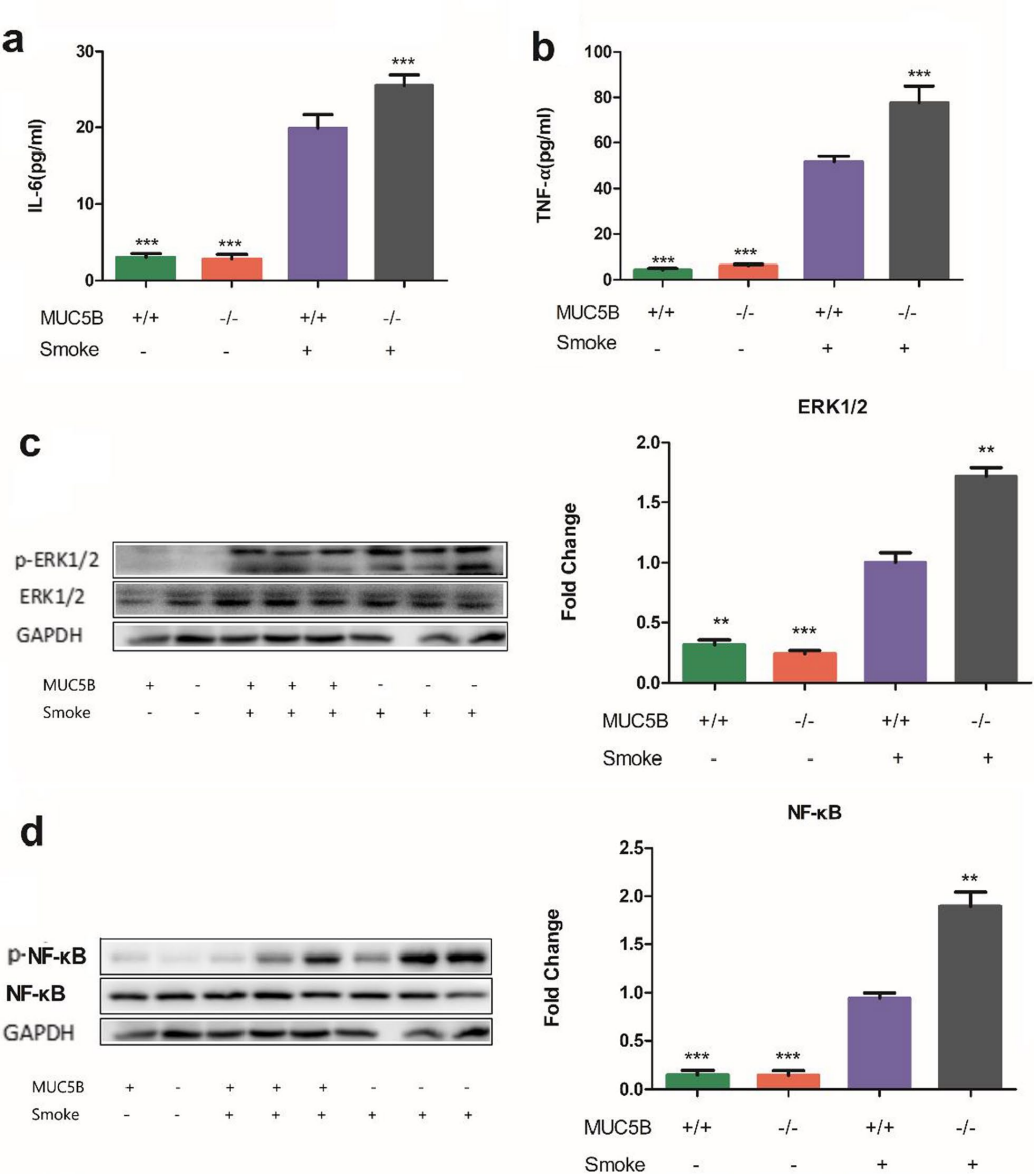
MUC5B^−/−^ mice have increased expression of macrophage-related inflammatory factors. MUC5B^+/+^ mice (n = 15 per group) and MUC5B^−/−^ mice (n = 10 per group). **a** BAL fluid IL-6 ELISA. **b** BAL fluid TNF-α ELISA. **c** ERK1/2 western blot and grayscale analysis. **d** NF-κB western blot and grayscale analysis. “*” indicates a significant difference; ***P < 0.001, **P < 0.01 compared to smoke-exposed MUC5B^+/+^ mice

Roy et al. showed changes in the function and morphology of pulmonary macrophages in MUC5B- mice [11]; thus, we examined the related inflammatory factors secreted by macrophages and associated pathways. There were significant increases in IL-6 (Fig. 4a) and TNF-α (Fig. 4b) in BAL fluid in mice exposed to smoke compared to control mice (P < 0.001). Among the mice in the smoke-exposed group, the levels of IL-6 and TNF-α were significantly increased in MUC5B^−/−^ mice compared with MUC5B^+/+^ mice (P < 0.001). The expression levels of ERK1/2 and NF-κB, regulators of inflammation and associated with macrophages, in the smoke-exposed group were increased compared with those in the control group (P < 0.001) (Fig. 3c, d). Moreover, among mice in the smoke-exposed group, the phosphorylation of ERK1/2 and NF-κB in lung tissue was higher in MUC5B^−/−^ mice than in MUC5B^+/+^ mice (P < 0.01).

### Worsened general conditions and lung function inMUC5B^−/−^ mice

**Fig. 5:**
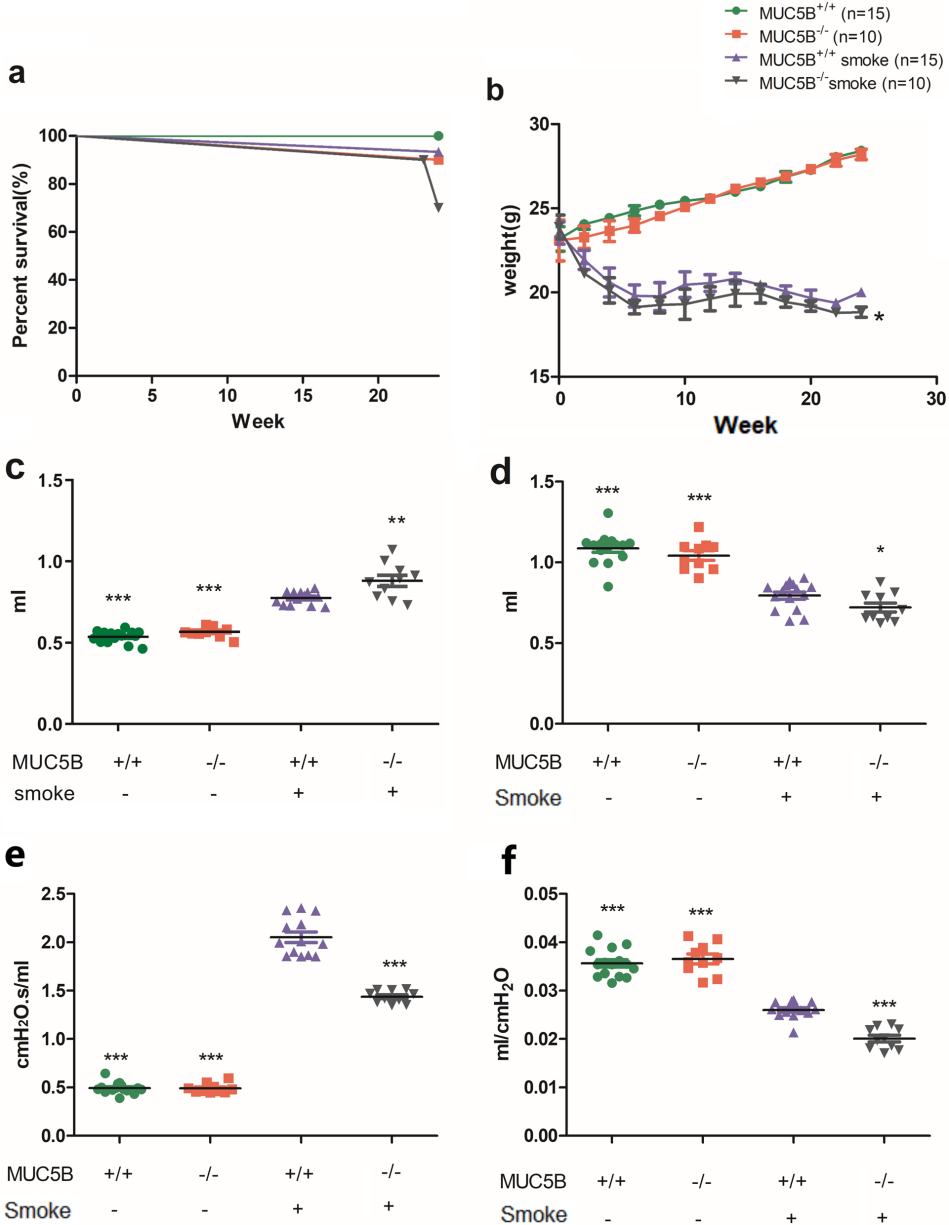
General conditions and lung function: MUC5B^+/+^ mice (n = 15 per group) and MUC5B^−/−^ mice (n = 10 per group). All tests were conducted after the end of model establishment. **a** Survival curves. **b** Weight change. **c** Functional residual capacity (FRC). **d** Forced expiratory volume in the first 100 ms (FEV100). **e** Airway resistance (RI). **f** Dynamic lung compliance (Cdyn). “*” indicates a significant difference; ***P < 0.001, **P < 0.01, *P < 0.05 compared to the smoke-exposed group of MUC5B^+/+^ mice

The mortality in the smoke-exposed group was higher than that in the control group, and in the smoke-exposed group, the mortality of MUC5B^−/−^ mice was higher than that of MUC5B^+/+^ mice (Fig. 5a). The body weights in the control group continued to increase throughout the experiment, while those in the smoke-exposed group continued to decrease, reaching the nadir at the 10th week and remaining low thereafter. Moreover, the body weights of MUC5B^−/−^ mice decreased significantly faster than those of MUC5B^+/+^ mice (P < 0.05) (Fig. 5b). In the smoke-exposed group compared with the control group, FRC (P < 0.001) (Fig. 5c) and RI (P < 0.001) (Fig. 5e) were significantly increased and FEV100 (P < 0.001) (Fig. 5d) and Cdyn (P < 0.001) (Fig. 5f) were significantly decreased. Among the mice in the smoke-exposed group, compared with MUC5B^+/+^ mice, MUC5B^−/−^ mice exhibited significantly lower RI (P < 0.001), Cdyn (P < 0.001) and FEV100 (P < 0.05) and significantly higher FRC (P < 0.001). In the control group, there was a trend of lower lung function in MUC5B^−/−^ mice than in MUC5B^+/+^ mice, but there was no significant difference (Fig. 5).

## Discussion

In this study, we showed, for the first time, that MUC5B can promote goblet cell differentiation and inflammation in a murine model of COPD model and that MUC5B exerts these effects by interfering with the STAT6-SPDEF pathway or the function of macrophages.

ES cell targeting was used to construct homozygous MUC5B knockout mice in our study (Additional file 1). In our study, the MUC5B^−/−^ control group was exposed to normal environment. Two of the MUC5B^−/−^ control group mice showed symptoms such as obvious respiratory distress, hunched posture, weight loss, and coughing. These two mice died at the 23rd week, while the others did not show a special phenotype or had only mild symptoms. These phenotypes are similar to those reported by Valque and Hélène et al. [22]. However, the MUC5B^−/−^ mice in Valque’s study were unable to reproduce, and a small number of mice without respiratory symptoms also showed pathological changes in the lungs. In our study, the lung function of the MUC5B^−/−^ control group was slightly worse than that of MUC5B^+/+^ control mice, and the inflammatory cell count was slightly higher in the former, but the differences were not significant. The difference between the studies may reflect the fact that the MUC5B knockout mice in Valque’s study were heterozygous, with differences in gene knockout methods; the difference may also be related to differences in sample size and breeding environment and individual differences in mice. However, in general, the construction of MUC5B knockout mice used in our study was successful.

The main finding of our study is that MUC5B regulates goblet cell differentiation. There are two main ways to quantify the differentiation of goblet cells in the airway: PAS staining or Alcian blue staining of lung pathology and detection of the goblet cell marker MUC5AC [23, 24], Under exposure to cigarette smoke, most of the secreted MUC5AC is secreted by goblet cells, and a small amount is secreted by submucosal glands [25, 26]. In our study, there was significantly less positive PAS staining in the lungs of MUC5B^−/−^ mice than in those of MUCB5^+/+^ mice (based on visual observation and quantitative PAS score) (Fig. 1a), and both the quantitative (ELISA, qPCR) and qualitative (immunohistochemical) results indicated lower MUC5AC expression in MUC5B^−/−^ mice (Fig. 1b, e, f). These findings indicate that the MUC5B^−/−^ mice had less differentiation of airway goblet cells than MUC5B^+/+^ mice in the development of COPD. We infer from these phenomena that MUC5B can promote airway goblet cell differentiation. As a small amount of goblet cells still existed in the airway of MUC5B^−/−^ mice, plus the secretion of submucosal glands, a certain amount of MUC5AC can still be detected. Additionally, the lower RI of MUC5B^−/−^mice was likely caused by the reduction in goblet cell differentiation (Fig. 5e), showing that MUC5B plays an important role in the differentiation of airway goblet cells in COPD. Other studies have also shown that MUC5B may regulate the differentiation of goblet cells. A study on muco-obstructive lung disease showed that after mucus hypersecretion modeling, severe mucus obstruction occurred in normal mice and MUC5AC^−/−^ mice, while mucus secretion in the airway of MUC5B^−/−^ mice was significantly reduced. Another study in an elastase mouse model yielded similar results [12, 13], indicating that muc5b can affect airway mucus secretion, while goblet cells are the main sources of airway mucus. These studies provide indirect support for our hypothesis.

SPDEF plays a major role in airway goblet cell differentiation [27]; it is positively regulated by STAT6 [23] and negatively regulated by FOXA2 [28]. Studies show that SPDEF is associated with MUC5B: in SPDEF^−/−^ mice, MUC5B expression has been found to be significantly reduced [15, 29], MUC5B expression has been found to be increased in neonatal mice overexpressing SPDEF [23], and SPDEF^**−/−**^ mice have phenotypes similar to those of MUC5B^**−/−**^ mice [15]. Therefore, the regulation of goblet cell differentiation by MUC5B in the development of COPD may be related to SPDEF. In our study, there was almost no expression of SPDEF in the lungs of MUC5B^−/−^ mice, which may be the main reason for the decrease in goblet cell differentiation in the lungs of these mice. To clarify the effect of MUC5B on SPDEF, we measured the expression of upstream factors of SPDEF. The results showed that in the lungs of MUC5B^−/−^ mice, the expression of STAT6, a promoter of SPDEF, was decreased, while the expression of FOXA2, an inhibitor of SPDEF, was increased; moreover, the levels of these factors were different from those in the control group. Previous studies have shown that the production of MUC5B is related to STAT6 and FOXA2 [30, 31]. STAT6 promotes MUC5B expression by binding with the transcriptional inhibitor FOXA2 at the mucin promoter [32, 33], and increased expression of FOXA2 can inhibit the expression of MUC5B [34]. Moreover, an increase in STAT6 or a decrease in FOXA2 leads to increases in mucus and goblet cell differentiation [27]. This evidence suggests that MUC5B can regulate SPDEF by promoting STAT6 expression and inhibiting FOXA2 expression, thus regulating the differentiation of airway goblet cells. However, since SPDEF can inhibit the expression of FOXA2, it is not clear whether MUC5B can directly inhibit the expression of FOXA2.

During the modeling period, MUC5B^−/−^ mice had higher mortality, faster weight loss and worse lung function than MUC5B^+/+^ mice. According to our experimental results, we hypothesize that these effects were caused by excessive inflammation related to the lack of MUC5B. Consistent with the findings of Roy et al. [11], the number of macrophages in MUC5B^−/−^ mice with a COPD-like phenotype was increased, and these cells showed abnormal functions (Fig. 2d). TNF-α, which is mainly produced by macrophages, was increased in MUC5B^−/−^ mice, and the downstream factors ERK1/2 and NF-κB, which are induced by TNF-α-mediated activation of the receptor TNFR1, were also increased (Fig. 4). Since TNFR1 can initiate the inflammatory response, MUC5B may reduce pulmonary inflammation by regulating the function of macrophages. Roy et al. showed that in MUC5B^−/−^ mice, the expression of anti-inflammatory factor IL-23, which is mainly secreted by macrophages, was significantly decreased, which supports our results. Consistent with our findings, the transmembrane mucin MUC1 can inhibit intestinal macrophage phagocytosis and the secretion of TNF-α after LPS stimulation, and MUC2 mitigates inflammatory responses in dendritic cells (DCs) of gut mucosa [35, 36]; in wild-type macrophages, MUC1 reduces the activation of NF-κB [37]. However, this effect does not depend on MUC1 expressed by the epithelium but on MUC1 expressed by macrophages [38], and Roy et al. showed that pulmonary macrophages do not express MUC5B. Therefore, the anti-inflammatory effect of MUC5B in the development of COPD is likely related to the function of macrophages.

Roy et al. [11] revealed the biological necessity of MUC5B in the airway, while our study aimed to determine the specific role of MUC5B and the underlying mechanism. The results of some studies suggest that excessive mucin concentrations are positively correlated with the severity of COPD, increased mucin production in COPD and decreased lumen fluid, which have detrimental effects on airway health [39, 40]; the increase in MUC5B in the sputum of COPD patients is associated with a decrease in lung function [5]. However, considering our results and previous results, the damage to the lungs may be due to the accumulation of inextricable mucus in the airway [41–44]; it might also be explained by the change in glycosylation (increased amounts of low-charge MUC5B) [5]. Therefore, the increase in MUC5B should be beneficial to the airway. Therefore, it is not advisable to remove a large amount of mucus or inhibit the expression of MUC5B for long periods. Properly promoting the expression of MUC5B in a controllable range to enhance airway defense can be a new therapeutic strategy for COPD. Some mucolytic agents, such as carbocysteine and N-acetylcysteine, can reduce the viscosity of mucus without affecting the expression of MUC5B, make it easier to expel mucus, and alleviate mucus obstruction. In addition, clinical studies have shown that the use of mucolytic agents can reduce the acute exacerbation rate of COPD [45, 46]. Moreover, our research provides a new target for the treatment of mucus hypersecretion; inhibiting STAT6 or promoting the expression of FOXA2 can effectively reduce mucus secretion, but the balance in mucus secretion should be considered.

However, our research lacks in vitro studies. Gas–liquid interface culture of primary airway epithelial cells in vitro and MUC5B overexpression studies are needed to confirm the role of MUC5B in regulating goblet cell differentiation.

## Conclusion

**Fig. 6:**
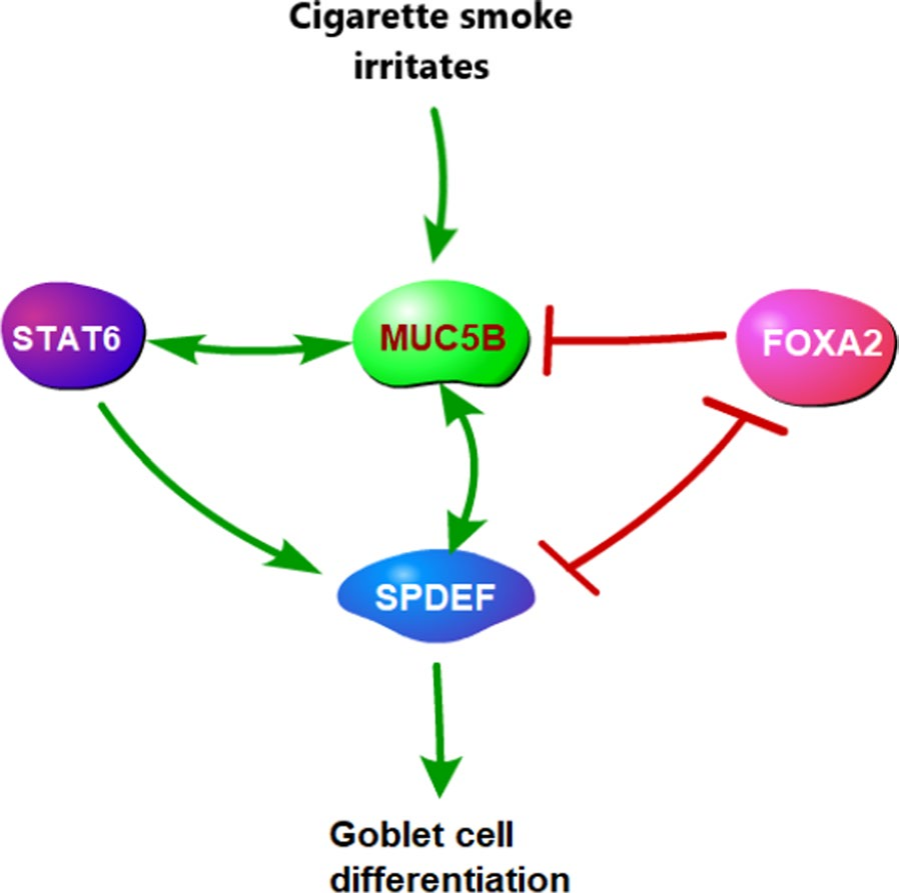
The cascade amplification process of goblet cell differentiation in the lung. Arrows indicate promotion, and line-capped lines indicate inhibition

Our study reveals the possible mechanism of mucus hypersecretion, described as follows: The production of pulmonary mucus is an amplification process in which MUC5B plays an important role (Fig. 6). When exposed to excessive, long-term stimulation (such as cigarette smoke), submucosal glands secrete MUC5B to promote the expression of STAT6 and SPDEF, which leads to goblet cell differentiation and mucus production. STAT6 can also promote the expression of MUC5B, strengthening the effects. FOXA2 acts as a negative regulator in this system.

In summary, our data show that MUC5B can regulate goblet cell differentiation by affecting the expression of SPDEF and that the role of MUC5B in regulating inflammation is related to the function of macrophages. MUC5B is a new candidate target for the treatment of mucus hypersecretion.

## Supplementary Information

**Additional file 1.** Construction of MUC5B knockout mice.

## Data Availability

All data generated or analyzed during this study are included in this published article [and its additional information files].

## Abbreviations

COPD: Chronic obstructive pulmonary disease
SPDEF: SAM-pointed domain-containing Ets-like factor
FOXA2: Forkhead box protein A2
FRC: Functional residual capacity
RI: Airway resistance
FEV100: Forced expiratory volume in 100 ms
Cdyn: Dynamic compliance
BAL: Bronchoalveolar lavage
HE: Hematoxylin–eosin
PAS: Periodic acid-Schiff
IHC: Immunohistochemistry
TNF-α: Tumor necrosis factor alpha
IL-6: Interleukin-6
NF-κB: Nuclear factor kappa-B
ERK1/2: Extracellular-regulated kinase 1/2
