# Supplementary material for: MUC5B regulates goblet cell differentiation and reduces inflammation in a murine COPD model
Source: Respir Res. 2022 Jan 18;23:11. doi: 10.1186/s12931-021-01920-8 (PMC8764756; doi:10.1186/s12931-021-01920-8)
Supplement: Supplementary file 1 — Additional file 1. Construction of MUC5B knockout mice. [file 12931_2021_1920_MOESM1_ESM.docx]

Construction of Muc5b gene flox mice.

Muc5b gene was modified by flox by targeting C57/B6J background ES cells. The brief process is as follows: the ES cell targeting vector was constructed by In-fusion method, which contains 2.705 kb 5 'homologous arm, 2kb flox region, PGK-Neo-polyA, 5.138kb 3' homologous arm and MC1-TK-polyA negative screening markers. After linearization, the vector was electro transfected into JM8A3 ES cells. After screening with G418 and Ganc, 192 resistant clones were obtained, and 10 positive clones with correct homologous recombination were obtained by long fragment PCR identification. The positive ES cell clones were amplified and injected into the blastocysts of C57BL/6J mice to obtain chimeric mice. Five positive F1 mice were obtained by mating with high proportion chimeric mice and Flp mice.


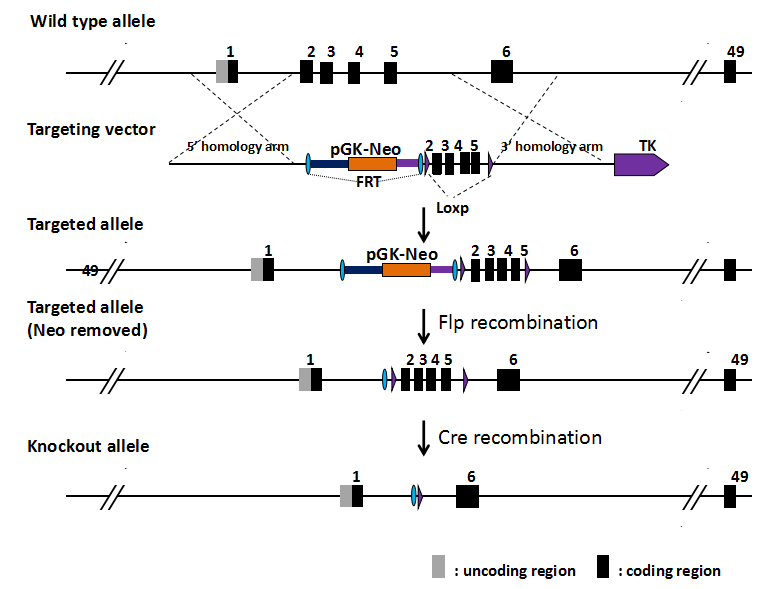


There was no obvious abnormality in flox heterozygous mice of Muc5b gene. After mating with the mouse DPPA3 Cre expressing Cre recombinant enzyme, the flox mouse obtained the whole-body knockout heterozygote Muc5b^+/-^; Cre^+/-^, and then mated the WT mouse, screened the whole-body knockout heterozygote muc5b^+/-^; and rearranged the heterozygote to mate with each other, and obtained the whole-body knockout homozygote muc5b^-/-^.

**Reproduction and identification of knockout mice**

**PCR**

| Primer | Sequence (5’→3’) | | | | Primer Type |
| --- | --- | --- | --- | --- | --- |
| P1 | TTCTGGCTGTCAGTCTGTGGAGGT | | | | Forward |
| P2 | GAGGGTCTGTGGTAGTCATGTGGG | | | | Reverse |
| PCR Reaction System | **Reaction Component** | | | | **Volume (µl)** |
|  | ddH2O | | | | 8.2 |
|  | 2×PrimeStar MAX mix* | | | | 10 |
|  | Primer I (10pmol/µl) | | | | 0.4 |
|  | Primer II (10pmol/µl) | | | | 0.4 |
|  | genomic DNA | | | | 1 |
|  | Total | | | | 20 |
|  | *PrimeStar MAX（TaKaRa，Code No：R045A） | | | | |
| Cycling Reaction | Step | Temp | Time | Note | |
|  | 1 | 94 °C | 3 min |  | |
|  | 2 | 94 °C | 30 sec |  |  |
|  | 3 | 56°C | 30sec |  |  |
|  | 4 | 72 °C | 2min30sec | 35 repeats to 2 | |
|  | 5 | 72 °C | 5min |  | |
|  | 6 | 12°C | Hold |  |  |
| Genotype | KO = ~414bp  Heterozygote = 414bp and 2245bp Wild type = ~2245bp | | | | |

**PCR results**


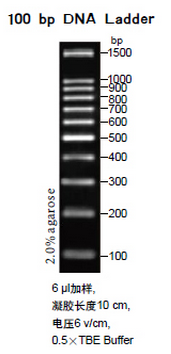

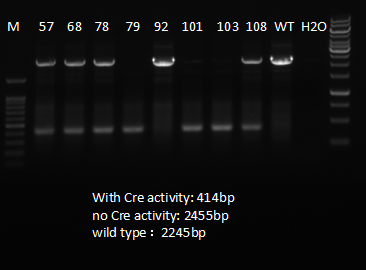

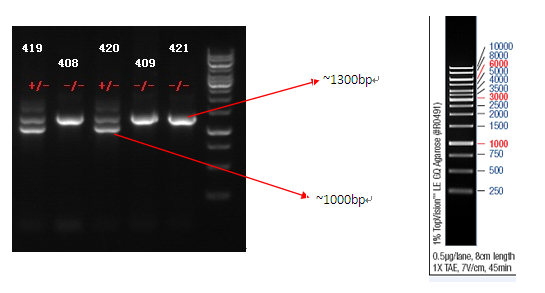


Fig. 1 Electrophoretic map of Muc5b KO PCR identification

WT: wild type mouse genome, H20: negative water control, Marker: 100bp DNA marker from Takara (3422A) & GeneRuler 1 kb DNA Ladder from Thermo Scientific (Cat No:SM0311)

Table 1: basic information table of mice

| mice ID | Muc5b KO |
| --- | --- |
| **57** | HE |
| **68** | HE |
| **78** | HE |
| **79** | HO |
| **92** | WT |
| **101** | HO |
| **103** | HO |
| **108** | HE |

HO: homozygous, HE: heterozygous, and WT is wild type.

**Identification**


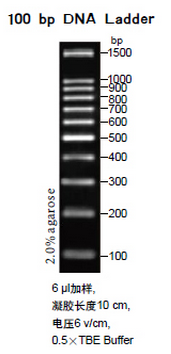

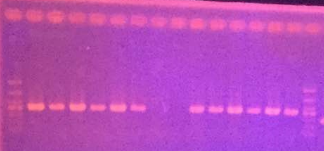


**H2O**

Muc5b KO PCR results

WT mice have two bands at 2245BP, heterozygous mice have a band at 2245BP and 414BP, and homozygous mice have bands only at 414BP.
